# MAP1203 Promotes *Mycobacterium avium* Subspecies *paratuberculosis* Binding and Invasion to Bovine Epithelial Cells

**DOI:** 10.3389/fcimb.2018.00217

**Published:** 2018-06-27

**Authors:** Jamie L. Everman, Lia Danelishvili, Lucero G. Flores, Luiz E. Bermudez

**Affiliations:** ^1^Department of Microbiology, College of Science, Oregon State University, Corvallis, OR, United States; ^2^Department of Biomedical Sciences, College of Veterinary Medicine, Oregon State University, Corvallis, OR, United States

**Keywords:** *Mycobacterium avium* subs. *paratuberculosis*, MAP1203, pathogen-host interaction, MDBK, RAW 264.7, binding, invasion

## Abstract

*Mycobacterium avium* subspecies *paratuberculosis* (MAP) is the causative agent of Johne's disease, chronic and ultimately fatal enteritis that affects ruminant populations worldwide. One mode of MAP transmission is oral when young animals ingest bacteria from the collostrum and milk of infected dams. The exposure to raw milk has a dramatic impact on MAP, resulting in a more invasive and virulent phenotype. The MAP1203 gene is upregulated over 28-fold after exposure of the bacterium to milk. In this study, the role of MAP1203 in binding and invasion of the bovine epithelial cells was investigated. By over-expressing the native MAP1203 gene and two clones of deletion mutant in the signal sequence and of missense mutations changing the integrin domain from RGD into RDE, we demonstrate that MAP1203 plays a role in increasing binding in more than 50% and invasion in 35% of bovine MDBK epithelial cells during early phase of infection. Furthermore, results obtained suggest that MAP1203 is a surface-exposed protein in MAP and the signal sequence is required for processing and expression of functional protein on the surface of the bacterium. Using the protein pull-down assay and far-Western blot, we also demonstrate that MAP1203 interacts with the host dihydropyrimidinase-related protein 2 and glyceraldehyde 3-phosphate dehydrogenase proteins, located on the membrane of epithelial cell and involved in the remodeling of the cytoskeleton. Our data suggests that MAP1203 plays a significant role in the initiation of MAP infection of the bovine epithelium.

## Introduction

*Mycobacterium avium* subspecies *paratuberculosis* (MAP) is a slow-growing pathogen, which causes Johne's disease in cattle and other ruminants. The bacterium is well described to readily change its phenotype during various stages of disease and upon interaction with a variety of environments. One method of MAP transmission to young animals is via contaminated colostrum and milk early in life. The mammary gland, its epithelial lining, and milk have all been shown to serve as a reservoir for MAP across the stages of the disease (Taylor et al., 1981; Sweeney et al., 1992; Stabel and Lambertz, 2004). Notably, MAP dramatically alters its phenotype when exposed to the hyperosmolar milk environment, resulting in a more virulent and infectious form of bacteria (Patel et al., 2006; Alonso-Hearn et al., 2010; Everman and Bermudez, 2015). The MAP1203 gene was identified with this phenotype, displaying expression over 28-fold upon incubation within raw milk (Patel et al., 2006), and suggesting potential role of this gene in invasive capabilities across the intestinal mucosa and in the pathogenesis of MAP.

Previous studies have identified several genes of MAP within the host involvement at the different stages of infection (Alonso-Hearn et al., 2008, 2010). It has been determined that the uptake of MAP by bovine intestinal epithelial cells requires the action of many proteins including the oxidoreductase MAP3464 and intracellular delivery of MAP3985 (Alonso-Hearn et al., 2008), which binds to cdc42 G-protein and induces cytoskeletal re-organization. The operon containing the intracellular persistence associated gene, *iip*A, plays a direct role in the invasion and survival of mycobacteria within the host (Gao et al., 2006). In addition, complementing the *Mycobacterium marinum iip*A gene knockout mutants with the homolog Rv1477 (*rip*A) gene of *Mycobacterium tuberculosis* (Mtb) confers increased binding, invasion and survival of *M. marinum* within macrophage cell lines (Gao et al., 2006). Studies have also found that the Mtb RipA protein, containing a conserved NLP60-peptidoglycan hydrolase domain, is important factor for remodeling of the cell wall during mycobacterial growth (Chao et al., 2013).

The fact is that MAP1203 is 79% homologous to the amino acid sequence of Mtb Rv1477, and contains the same NLP60 peptidase and RGD-binding domain, and the signal sequence as the *iip*A gene of *M. marinum* (Gao et al., 2006). In this study, we aimed to understand and functionally characterize MAP1203 during MAP invasion and infection of bovine epithelial cells. By utilizing the deletion constructs of the RGD integrin-binding domain and the protein signal sequence, here, we demonstrate the role of MAP1203 in the initiation of infection and invasion of MDBK cells.

## Materials and methods

### Bacterial culture

*Mycobacterium avium* subspecies *paratuberculosis* strain K-10 (ATCC BAA-968) was cultured at 37°C on 7H10 agar (BD; Franklin Lakes, NJ) supplemented with casein hydrolysate (1 g/L; BD), 10% (vol/vol) oleic acid, albumin, dextrose, and catalase (OADC; Hardy Diagnostics; Santa Maria, CA), and ferric mycobactin J (2 mg/L; Allied Monitor, Fayette, MO) for 3–4 weeks prior to experiments. *Mycobacterium smegmatis* (MS) strain mc^2^155 (ATCC) was cultured on 7H10 agar supplemented with 10% OADC at 37°C. For experimental suspensions, bacteria were suspended in HBSS, passaged through a 23-gauge needle 5 times and clumps allowed to settle for 10 min. The top 50% of the suspension was used for the inoculum preparation (Patel et al., 2006).

### Mammalian cell culture

Madin-Darby bovine kidney (MDBK) epithelial cells (CCL-22) and RAW 264.7 macrophage cultures (TIB-71) were obtained from the American Type Culture Collection (ATCC; Manassas, VA). Both cells lines were cultivated in Dulbecco's Modified Eagle's Medium (DMEM) supplemented with 10% heat-inactivated fetal bovine serum (FBS; Gemini Bio-Products; West Sacramento, CA), at 37°C in 5% CO_2_.

### Construction and overexpression of deletion and mutated clones of MAP1203 in *M. smegmatis* and MAP

We chose to create dominant negative clones of MAP to characterize domains of MAP1203 gene and study how mutations in a particular domain would impact the gene function and impair bacterial fitness. We utilized the constitutive and inducible expression vector pJAM2 (Triccas et al., 1998), and inserts mutations in the motif-known region of the gene, which becomes the predominant gene (*vs* the wild-type gene) because it is constitutively expressed. In general, mycobacteria generate approximately 1–3 copies of the plasmid per cell. The MAP1203 gene (1,410 bp) was PCR amplified from the MAP genomic DNA using 1203XbaIFwd and 1203XbaIRev primers listed in the Table 1, and as follows: 95°C for 5 min, 35 cycles of 95°C for 1 min, 60°C for 30 s, 68°C for 2 min, followed by 68°C for 10 min. Mutations in the MAP1203 gene were introduced using the Splicing by Overhang Extension (SOE) PCR. The RCE mutation was created by changing RGD amino acid (aa) sequence to an RCE at the 417 aa residue of MAP1203. The Δ Signal Sequence (ΔSS) mutation was created by eliminating 16–39 aa residues of the predicted signal sequence cleavage site (Petersen et al., 2011). RCE fragments were PCR amplified using the primer pairs 1203XbaIFwd/MAP1203_RGDmu_B and 1203XbaIRev/MAP1203_RGDmu_C, while the ΔSS fragments were amplified using the primer pairs 1203XbaIFwd/MAP1203_SSmuB and 1203XbaIRev/MAP1203_ SSmuC with the PCR program described above. Equimolar amounts of fragments for each mutant were incubated together for 10 min at 50°C before PCR amplification as follows: 95°C for 5 min, 40 cycles of 95°C for 1 min, 60°C for 1 min, 68°C for 2 min, followed by 68°C for 10 min. PCR fragments were cloned into the XbaI site of the acetamide inducible plasmid pJAM2 (Triccas et al., 1998) resulting in pJAM2:MAP1203, pJAM2:RCE and pJAM2:ΔSS constructs.

**Table 1:** Primers used to construct the MAP1203 dominant negative expression vectors.

**Primer Name**	**Sequence (5′-3′)**
1203XbaIFwd	gtgtctagaatgagacgcacacgctgg
1203XbaIRev	gtgtctagaccactcgatgtaacggacc
MAP1203_RGDmu_B	gatgacttcgcagcggcgcat
MAP1203_RGDmu_C	atgcgccgctgcgaagtcatc
MAP1203_SSmu_B	ggcgacgggatccctcgcggccggccgcgcggaag
MAP1203_SSmuC	ccggccgcgagggatcccgtcgccgacaacctgg
MAP1203_pETHindIII_Fwd	gtgaagcttaagaatgagacgcacacgctgg
MAP1203_pETEcoRI_Rev	gtggaattcccactcgatgtaacggacc

To prepare electropcompetent cells, either *M. smegmatis* or MAP grown to the mid-log phase were washed four times with 10% glycerol and 0.1% tween-80, centrifuged at 2,000 × g for 15 min each time and the final bacterial pellets were suspended in 10% glycerol. Aliquots of 200 μl of each culture were electroporated in a 0.2 cm cuvette at 2.5 kV, 1,000 ohms, and 25 μF. *M. smegmatis* cultures were recovered in 7H9 broth supplemented with 10% OADC for 3 h at 37°C with shaking at 200 rpm and plated onto 7H10/OADC supplemented with kanamycin (50 μg/ml). MAP cultures were recovered in 7H9 broth supplemented with 10% OADC and mycobactin J (2 mg/L) for 24 h at 37°C prior to plating cultures on 7H10/casein hydrolysate/OADC/mycobactin J and kanamycin (400 μg/ml). Plate-grown MAP colonies were photographed under 40x magnification and the area of each colony was measured using ImageJ software (Schneider et al., 2012).

To induce the expression of MAP1203, *M. smegmatis* cultures were grown to an OD_600_ 0.5 and induced by the addition of 0.2% acetamide and 0.2% succinic acid for 6 h at 37°C at 200 rpm. MAP cultures were grown to an OD_600_ 0.3 and induced with 0.2% acetamide and 0.2% succinic acid for 24 h at 37°C in stationary culture. Proteins were extracted and analyzed using a rabbit polyclonal anti-MAP1203 antibody (1:300) in 4% nonfat dry milk as primary probe, and detected using a goat anti-rabbit IR800 secondary antibody (1:10,000) (Licor).

### Invasion and survival assays

MDBK or RAW 264.7 cells were seeded into 48-well plates and grown to 80% confluence prior to experiments. Cells were infected at an MOI of 10:1 with bacterial inoculum resuspended in DMEM culture medium. The infection was synchronized at 220 × g for 5 min prior to incubation for 2 h at 37°C with 5% CO_2_. Monolayers were washed three times with HBSS and incubated for 2 h in DMEM supplemented with amikacin (200 μg/ml) to kill extracellular bacteria (Bermudez and Young, 1994). To quantify the bacterial uptake, cells were lysed after amikacin treatment with 0.1% triton X-100 in deionized water. For survival assays, infected monolayers were incubated for additional 14 h prior to lysis. Lysates were collected, serially diluted and plated for Colony Forming Unit (CFU) quantification.

### Purification of MAP1203

The native MAP1203, RCE and ΔSS were re-amplified from pJAM2 constructs using MAP1203_pETHindIII_Fwd and MAP1203pETEcoRI_Rev primers (Table 1) and the program described above. Fragments were cloned into the HindIII/EcoI restriction sites of the pET6xHN-N-terminal expression vector. Resulting constructs were transformed into *E. coli* BL21 DE3 (Life Technologies) and grown on LB agar plates with 100 μg/ml ampicillin. *E. coli* colonies grown to OD_600_ 0.3 were inoculated into 250 ml LB broth supplemented with ampicillin at 37°C at 200 rpm. Cultures were induced by the addition of 1 mM IPTG (Sigma-Aldrich) for 3 h prior to collection of the bacterial pellet for protein analysis. Bacteria were lysed using the xTractor Buffer (Clontech) as per manufacturer's instructions, separated on the SDS-PAGE protein gel, probed for 6xHN-tagged MAP1203 expression using a goat anti-6xHN primary antibody (1:2,000; Clontech Laboratories, Inc), and visualized with a rabbit anti-goat IR800 secondary antibody (1:10,000) (Licor). The MAP1203 protein was purified on the nickel columns as per manufacturer's instructions (Clontech).

### Bacterial-host protein interaction studies

The *E. coli* clone overexpressing MAP1203 and the control clone containing empty pET6xHN-N vector were mechanical disturbed in bead-beater. The lysed bacterial samples were cleared by centrifugation and filtration through 0.2 μm filter. MDBK cells were harvested from the 75 mm^2^ flask, washed, and mechanically lysed in PBS supplemented with protease inhibitor cocktail (Sigma-Aldrich). Mammalian cell lysate was cleared by centrifugation at 60 × g for 10 min followed by filtration through 0.2 μm filter. The total protein extract from control or MAP1203 expressed *E. coli* were loaded on to columns of His60 nickel resin and washed per manufacturers protocol. Prior to the elution step, the total protein extract from MDBK cells were loaded with the immobilized MAP1203 on the nickel column and incubated overnight at 4°C with rotation. The following day, samples were washed according to the manufacturer's protocol (Clontech) to remove unbound host cell proteins and then eluted. The concentrated elutes were mixed with an equal volume of 2X Laemmli sample buffer (Bio-Rad), resolved on a 12% Mini-Protean precast SDS-PAGE protein gel (Bio-Rad) and stained with Coomassie blue. The proteins of interest were excised and processed using In-Gel digestion (Thermo Scientific). Proteins were reduced and alkylated via incubation with equal amounts of DTT and iodoacetamide at a final concentration of 10 mM, and then trypsin digested in solution at 37°C for 5 h. The mass spectrometric sequencing by electrospray ionization mass spectrometry (ESI-MS/MS) and data analysis was performed at the Oregon State University mass proteomics (OSU) facility.

Concurrently, the far-Western blot was performed for identification and confirmation of host cell surface proteins. Briefly, surface proteins of MDBK cells were labeled with the sulfo-NHS- LC biotin (Thermo Scientific), washed, and harvested from the 75 mm^2^ flask as previously described (Danelishvili et al., 2007). Cells were lysed and biotin-labeled proteins were recovered with streptavidin-labeled magnetic beads (Promega). The sample was boiled in Laemmli sample buffer and separated onto SDS-PAGE gel, transferred to nitrocellulose membrane, and blocked with 3% bovine serum albumin (BSA). Membranes were probed with purified 6xHN:MAP1203 protein for 4 h, washed, and visualized with anti-6xHN rabbit antibody and corresponding IRDye secondary antibody (Li-Cor Biosciences, Inc) at a dilution of 1:5,000 for 30 min. Proteins of interest were detected using an Odyssey Imager (Li-Cor). The MAP1203-interacting host proteins were compared from both studies.

### Statistical analysis

The data analysis was performed by comparing the experimental groups using the Student's *t*-test and ANOVA. *P*-values < 0.05 were considered significant.

## Results

### The role of MAP_1203 protein domains during infection

**Figure 1:**
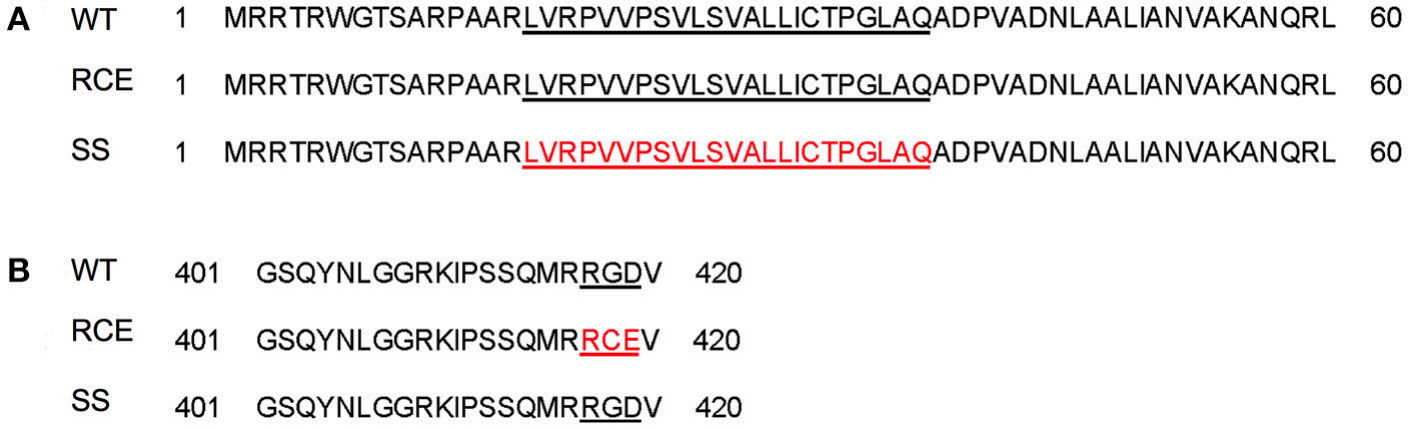
**(A)** MAP clones of the signal sequence deletion and of missense mutations replacing the integrin motif from RGD into RDE in MAP1203. A signal sequence deletion and RCE mutation clones were constructed to identify the role of each domain of MAP1203. **(B)** Underlined regions indicate sequence of interest, and amino acids in red indicate the sequence deleted or mutated from the native MAP1203 gene. WT, (wild-type) native gene; RCE, mutated RGD binding domain; SS, signal sequence.

To determine the role of MAP1203 during infection and interaction with both epithelial and macrophage cells, we first analyzed if there were homologous genes in closely related organisms and determined the presence of domains of interest within the hypothetical MAP1203 sequence. At both the nucleotide and amino acid levels, MAP1203 is highly homologous to *M. marinum* MMAR_2284 encoding an invasion and intracellular persistence protein (IipA) and to *M. tuberculosis* Rv1477 encoding the protein RipA (Table 2). The amino acid sequence at the C-terminus of the MAP1203 protein contains the arginine-glycine-aspartic acid (RGD) peptide motif (Figure 1), a well described integrin-binding domain used by viruses and bacteria to bind to and initiate the invasion process of the host cells (Bomsel and Alfsen, 2003; Asokan et al., 2006; Zimmermann et al., 2010). Analysis of putative signal sequences and the cleavage site was performed using SignalP 4.0 (Petersen et al., 2011), and indicates that a predicted signal sequence is present at the N-terminus of the protein with a cleavage site between residue 39–40 (Figure 1).

**Table 2:** MAP1203 sequence homology.

**Organism**	**Gene**	**Protein**	**Nucleotide homology (%)**	**Amino acid homology (%)**	**Function**
MAP	MAP1203	MAP1203	–	–	Hypothetical
*M. marinum*	MMAR_2284	IipA	79	75	Invasion and intracellular persistence
*M. tuberculosis*	Rv1477	RipA	81	79	Unknown, secreted, predicted virulence protein

To characterize the MAP1203 protein and dissect the function of identified domains, we created dominant-negative vector constructs to over-express identified domains *in vitro*. We mutated the RGD domain found at residues 417–419 in the amino acid sequence of MAP1203 to an RCE sequence (Figure 1), known to abrogate the binding effect to host cell integrin molecules (Lu et al., 2003; Chen et al., 2009; Zimmermann et al., 2010). To interrupt the function of the signal sequence we deleted amino acid residues at 16–39 location, resulting in a truncated signal sequence and interrupted cleavage recognition site in the protein. The changes in RCE and ΔSS domain sequences were verified and cloned into the acetamide-inducible plasmid pJAM2 for further analysis of function.

### Overexpression of MAP1203 in *M. smegmatis* influences infection of both epithelial cells and macrophages

**Figure 2:**
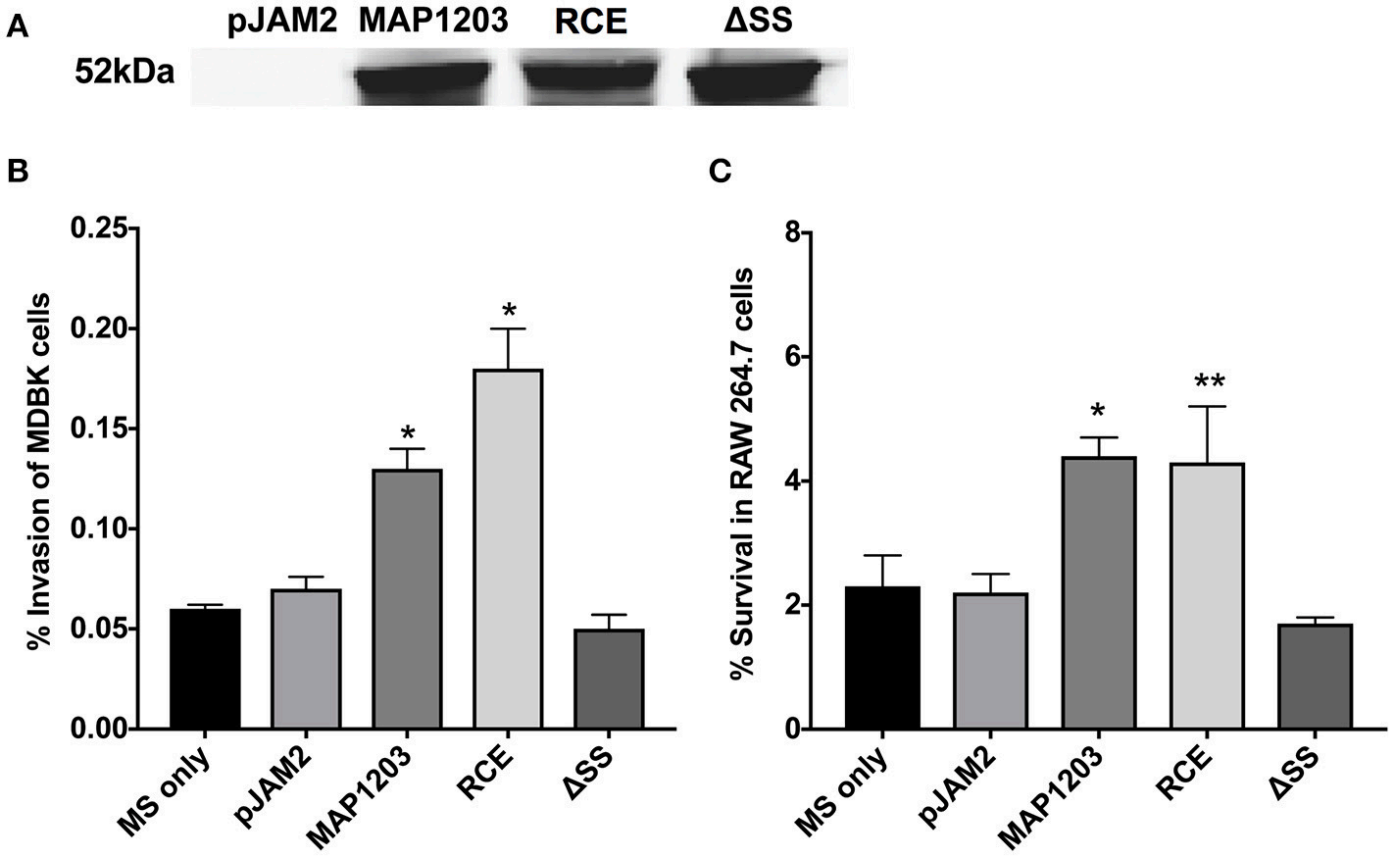
MAP1203 overexpression in *M. smegmatis*. **(A)** Cultures of *M. smegmatis* clones containing the control empty plasmid pJAM2, pJAM2:MAP1203, pJAM2:RCE, or pJAM2:ΔSS were grown to an OD_600_ 0.5 and then induced by adding 0.2% acetamide and 0.2% succinic acid to the medium for 6 h at 37°C at 200 rpm. Bacteria were mechanically disrupted in the bead-beater and cleared lysates were separated on the SDS-PAGE gel for Western blotting of the MAP1203 protein (52 kDa). The blot shown is representative of the expression of constructs for all *M. smegmatis* assays completed. **(B)** MDBK epithelial cells were infected with the induced clones of *M. smegmatis* for 2 h and percentage of invasion was calculated from the original inoculum used to infect the bovine cells. ^*^p < 0.05, significance compared to *M. smegmatis* with and without pJAM2 vector. **(C)** The RAW 264.7 macrophages were incubated with the induced clones of *M. smegmatis* and after 2 h extracellular bacteria were removed from cells by washing and antibiotic treatment. After 14 h of infection, macrophages were lysed and intracellular bacterial were quantified by Colony Forming Unit counts. The percentage of survival was calculated from the CFU counts of intracellular bacteria that entered RAW 264.7 cells during 2 h invasion. Data represent the mean ± SEM of three independent experiments conducted in triplicate. ^*^p < 0.05, ^**^p < 0.01 significance compared to *M. smegmatis* with and without pJAM2 vector as determined by Student's *t*-test.

To understand the function of MAP1203 during mycobacterial infection we utilized the fast-growing *M. smegmatis* as a surrogate for slow-growing MAP in our preliminary experiments. The *M. smegmatis* strain mc^2^ 155 does not contain the MAP1203 homologous gene. The fast-growing mycobacterial species are highly amenable to transformation and genetic manipulations, and it provides quick insight into the function of proteins of interest during initial investigation. Thus, we transformed each of our constructs into *M. smegmatis* and the native MAP1203 (wild-type), RCE and ΔSS proteins were successfully expressed after 24 h of induction of *M. smegmatis* cultures (Figure 2A).

Bovine MDBK epithelial cells were infected with *M. smegmatis* clones over-expressing the wild-type MAP1203 gene and mutated RCE and ΔSS constructs for 2 h prior to quantification of intracellular bacteria. Over-expression of the wild-type protein significantly increased the invasion capability of the naturally non-invasive *M. smegmatis* (Figure 2B). The RCE mutant displayed the same invasion ability as the wild type, while the signal sequence deletion mutant appears to be unable to confer the invasion advantage to *M. smegmatis* (Figure 2B). These data indicate that MAP1203 plays an important role in the initial interaction with the host epithelial cells during bacterial invasion. On the other hand, neither the wild-type MAP1203, nor RCE and ΔSS mutant constructs altered the level of bacterial uptake by macrophages after 2 h of infection compared to the strain carrying the empty expression vector (data not shown).

We next analyzed the influence of MAP1203 expression on the ability of *M. smegmatis* to survive within murine RAW 264.7 macrophages. Murine macrophages are readily used to study interactions between MAP and the phagocytic host. The intracellular survival assay of *M. smegmatis* expressing the wild-type MAP1203, RCE, and ΔSS proteins suggests that MAP1203 promotes intracellular survival of MAP within RAW 264.7 macrophages. The survival rate of *M. smegmatis* expressing the wild-type MAP1203 and RCE was greater than 2-fold higher than that of bacterial populations expressing the MAP1203 ΔSS construct or the empty pJAM2 vector after 14 h of infection (Figure 2C). Our data indicate that the RGD domain does not serve as a ligand for initial binding of the bacterium to the integrin receptors on the macrophage membrane, and that the signal sequence is required for the production or complete function of MAP1203 during murine macrophage infection.

### The role of MAP1203 during MAP infection of epithelial cells

**Figure 3:**
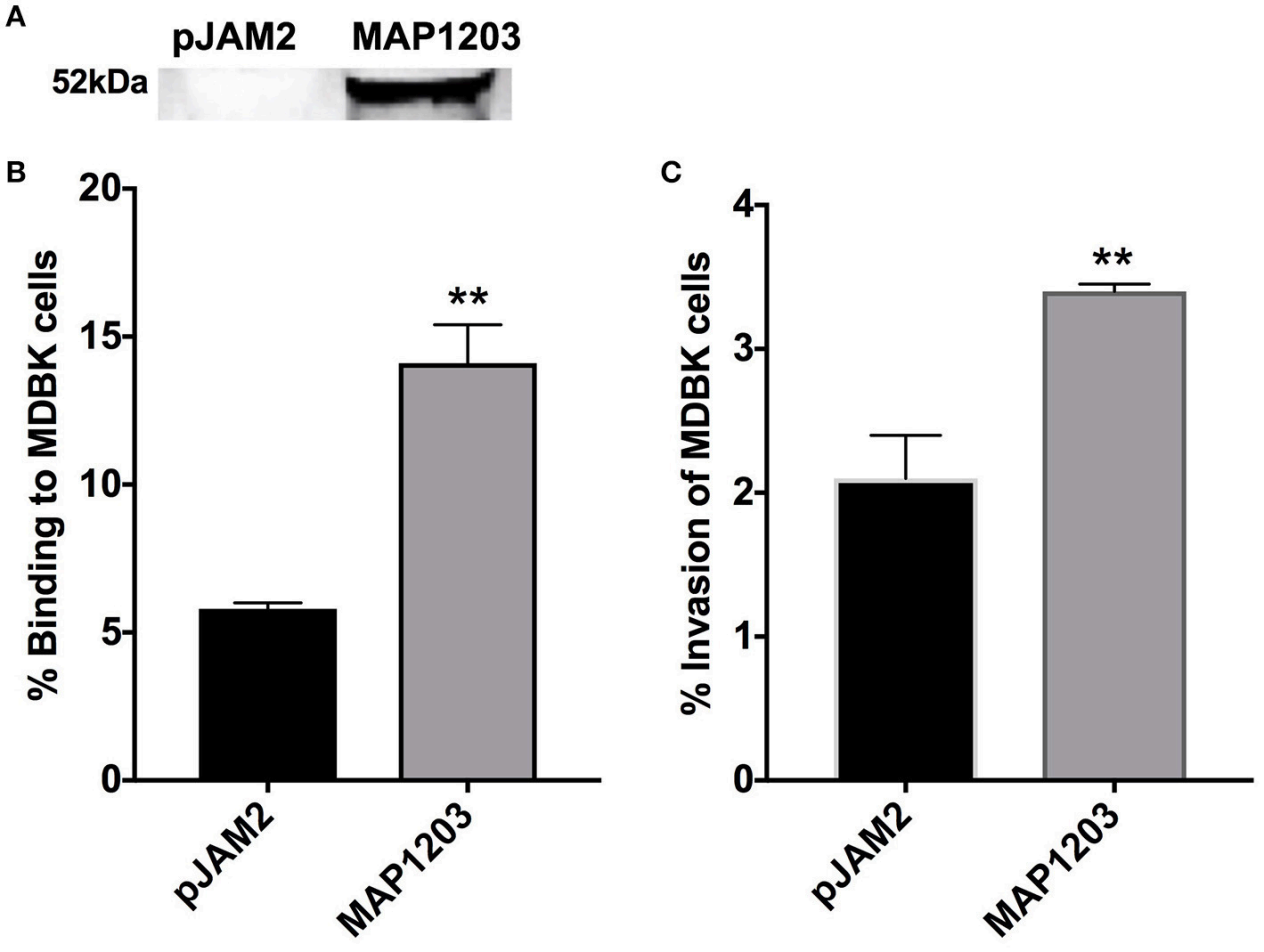
Role of MAP1203 in MAP binding and invasion of bovine MDBK epithelial cells. **(A)** MAP cultures expressing either control pJAM2 vector or MAP1203 protein were analyzed by Western blotting. The image is representative of each experiment conducted in the MAP expression studies. MAP containing either the empty pJAM2 vector or the pJAM:MAP1203 plasmid was used to infect MDBK cell monolayers to assess the role of MAP1203 in the binding **(B)** or invasion **(C)** of bovine MDBK epithelial cells after 2 h infection. Data represent the mean ± SEM of 4 independent experiments each conducted in triplicate. ^**^p < 0.01 significance compared to the MAP clone containing the control plasmid.

Using the surrogate *M. smegmatis* can offer relatively rapid insight into the function of a protein, but does not provide the complete genetic and physiologic response of the original bacteria from which it was identified. Thus, we transformed pJAM2 control and pJAM2:MAP1203 construct into MAP and the wild-type MAP1203 was successfully expressed after 24 h of induction of MAP cultures (Figure 3A).

MDBK epithelial cells were infected with induced strains of MAP for 2 h at 4°C to assess binding capabilities, and at 37°C to investigate the invasion rate of each culture. MAP culture over-expressing the wild-type MAP1203 protein is able to bind to the surface of epithelial cells nearly three times more than control cultures with the empty pJAM2 vector (Figure 3B). Additionally, the MAP1203 overexpressing MAP clon e is able to invade MDBK epithelial cells at a significantly higher level than the control clone (Figure 3C). These data clearly indicate that MAP1203 plays a significant role in the initiation of infection between MAP and bovine epithelial cells as it is able to enhance both the binding and invasion ability of MAP during early infection.

### Observational and experimental evidence that MAP1203 most likely is a surface associated protein

The MAP1203 homologous protein IipA of *M. marinum* has been described as an important host invasion factor for mycobacterial species (Gao et al., 2006). Likewise, the MAP1203 homologous *M. tuberculosis* protein encoded by Rv1477 is a predicted membrane surface associated protein (Chao et al., 2013). In order for a protein to impact the pathogens binding ability to the host cells, this factor would need to be expressed on the cell surface to come in contact with the host cell receptors or surface proteins and facilitate invasion and uptake of invading microorganism. MAP1203 has not been shown to be a surface associated protein; however, this gene is highly upregulated upon exposure to the milk environment, and through a series of experimental observations, suggesting that MAP1203 most likely is a surface associated protein.

**Figure 4:**
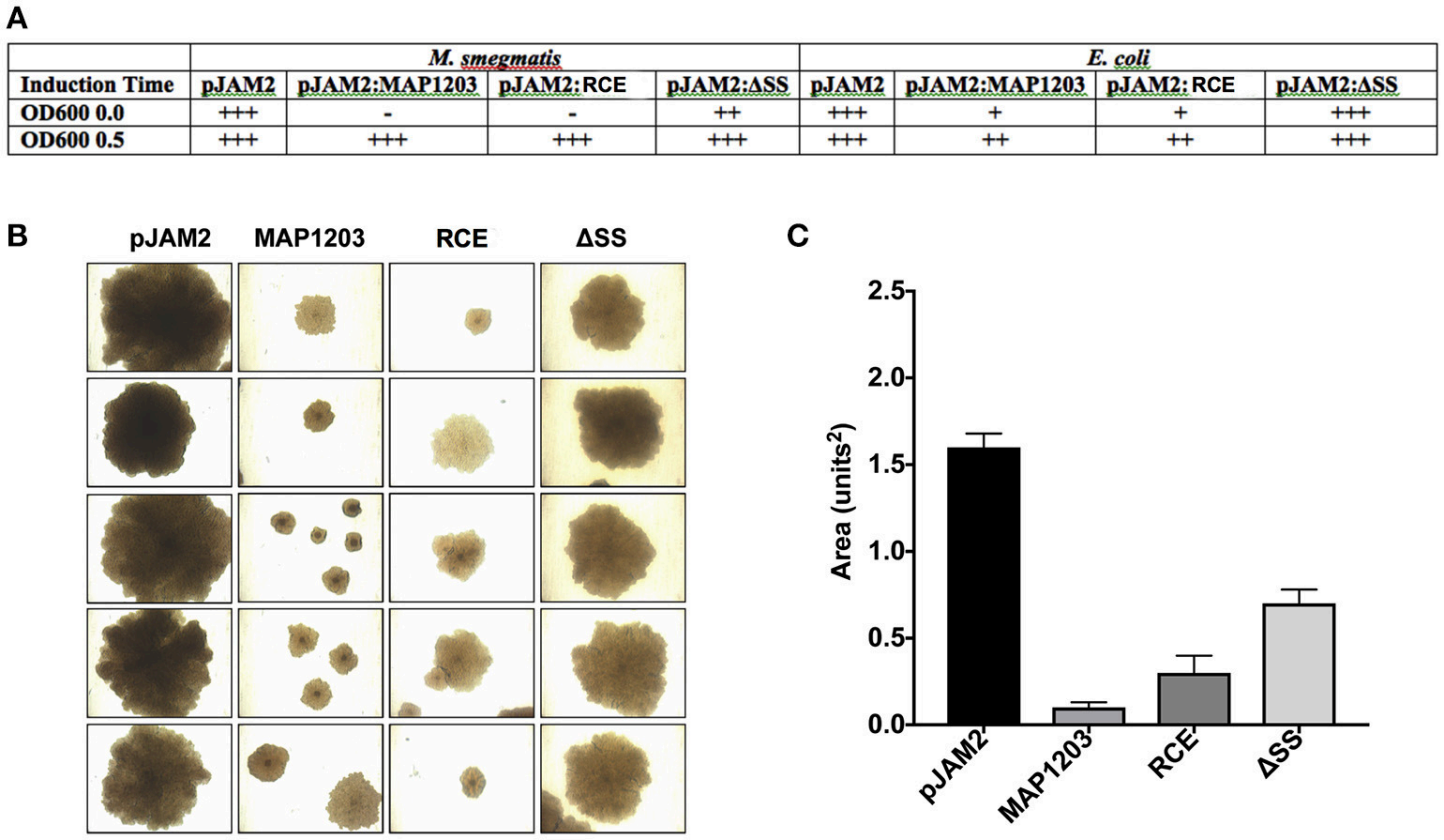
Toxicity of MAP1203 protein. *M. smegmatis, E. coli* and MAP were electroporated with pJAM2, pJAM2:MAP1203, pJAM2:RCE, or pJAM2:ΔSS constructs. **(A)** Observational growth characteristics of *M. smegmatis* and *E. coli* cultures were recorded. **(B)** MAP colonies were photographed under 40x magnification using the light microscope. **(C)** The area of each colony was measured using the ImageJ software. Images and data represent measurements taken for 20 individual colonies.

It is described that often the cell wall proteins, when over-expressed, result in bacterial toxicity or lysis (Montigny et al., 2004). We note that the long-term over-expression of MAP1203 is toxic (Figure 4). The induction of MAP1203 protein synthesis in *M. smegmatis* immediately at the time of inoculation (rather than at OD_600_ 0.5) we can conclude that the over-expression of MAP1203 is lethal to *M. smegmatis* and results in no bacterial growth over a 5-day time period, monitored with the optical density (Figure 4A). When we express MAP1203 constructs in pET6xHN_N vector of *E. coli* strain BL21 pLysS (meant to silence background expression of toxic proteins), we observe that expression of the wild-type MAP1203 and RCE is toxic. *E. coli* does not grow well in culture and the optical density decreases by half after 3 h induction, indicating the bacterial cell lysis. The mutation in the signal sequence (ΔSS) does not have any effect on the bacterial growth (Figure 4A). Lastly, we have observed that the MAP cultures containing either the control pJAM2 vector alone, or the pJAM2 vector constructs of the wild-type MAP1203, RCE, or ΔSS produce different colony size over the same period of incubation, suggesting that MAP1203 may have an effect on the growth rate or ability of MAP to grow in culture (Figures 4B,C).

### MAP1203 interacts with the surface proteins of bovine epithelial cells

**Figure 5:**
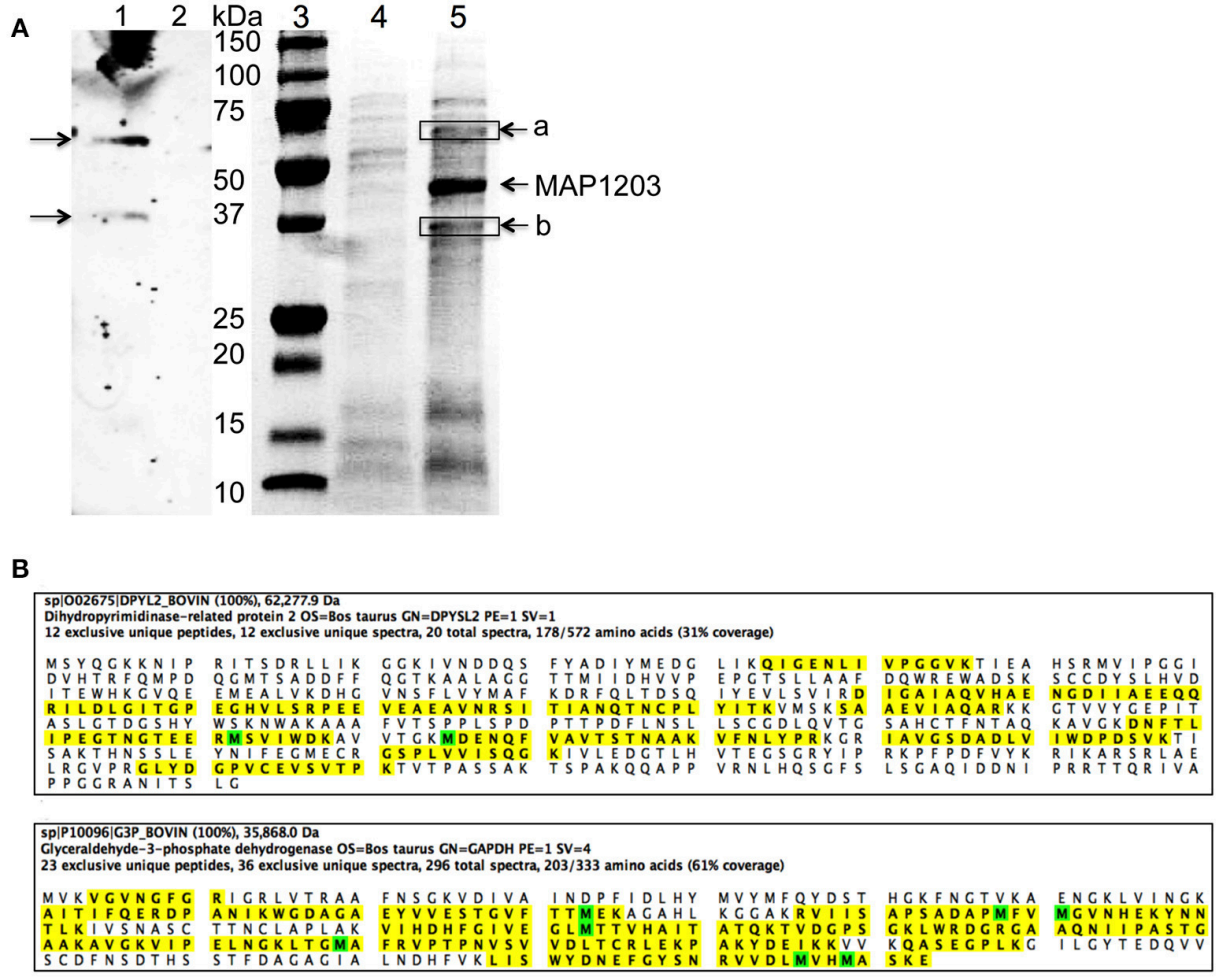
The host proteins interacting with MAP1203. **(A)** Identification of potential MAP1203 binding host partners by far-Western blotting and pull-down assays. (Lane 1) The biotin-labeled surface proteins of MDBK cells were transferred to a nitrocellulose membrane and incubated with the recombinant MAP1203 (0.5 mg) overnight at 4°C on the shaker. Bound proteins were visualized with 6xHN antibody as described in the materials and methods; (Lane 2) The control membrane (without any host proteins) was subjected to same procedures as lane 1; (Lane 3) Molecular Weight, Protein molecular marker. (Lane 4) The control pull-down assay was performed with *E. coli* lysate that contained the empty overexpression vector, and was exposed to total proteins of MDBK cells. Bands visualized with Coomassie staining represent non-specific host proteins that were bound to the column; (Lane 5) The cleared sample of *E. coli* overexpressing MAP1203 protein was incubated with the total protein lysate of bovine epithelial cells and unique proteins identified in the experimental lane (a and b) were excised and sequenced. **(B)** The MAP1203 bound host proteins were identified as Dihydropyrimidinase-related protein 2 and Glyceraldehyde 3-phosphate dehydrogenase; the identified peptides by MS are marked in yellow; Oxidized Methionine (M) is marked in green.

We demonstrate above that MAP1203 may serve as a cell wall protein involved in the binding to and invasion of MDBK bovine epithelial cells. To further understand the role of MAP1203 during infection, we conducted protein-protein interaction studies to identify the host proteins that directly interact with MAP1203 on epithelial cell surface. We conducted protein pull-down assay and a far-western blotting (Figure 5A). The mass spectrometric analysis of unique bands visualized and present in both assays identified the host Dihydropyrimidinase-related protein 2 (DPYL2) and Glyceraldehyde 3-phosphate dehydrogenase (G3P; Figure 5B). Glyceraldehyde 3-phosphate is a protein associated with distinct functions including glucose metabolism of the cell and cell wall-associated protein trafficking (Gozalbo et al., 1998). It also has been identified as a cell membrane protein associated with the attachment in eukaryotic organisms (Gozalbo et al., 1998).

## Discussion

In the mammary gland, while exposed to the milk, MAP undergoes phenotypic changes that increase the ability of the pathogen to efficiently infect mucosal epithelial cells (Patel et al., 2006; Alonso-Hearn et al., 2010). The manner by which MAP enters the animal body has been extensively investigated. The specialized epithelial M cells are one of the host cells that bacteria interacts to cross the intestinal barrier (Secott et al., 2004). Additional studies have identified intestinal enterocytes that MAP uses to translocate across the intestinal wall (Bermudez et al., 2010; Bannantine and Bermudez, 2013; Ponnusamy et al., 2013). It seems that both M cells and enterocytes may serve as routes of entry for the bacterium, but whether the route has any impact on the outcome or evolution of disease is still unknown.

Interaction with epithelial cells of the gastrointestinal tract is the important initial step for MAP to cross the mucosal layer. Past studies have demonstrated that after MAP attaches to the mucosal epithelial cells, it requires an oxidoreductase protein to trigger the secretion of MAP3985 factor initiating the uptake of the bacteria by the cytoskeleton reorganization (Alonso-Hearn et al., 2008). If there is an opportunity to develop a mucosal vaccine to prevent MAP infection in the first place, additional information is required on molecular mechanisms how the bacteria binds to the host cell.

The MAP1203 gene ortholog in *M. tuberculosis* (*rip*A) is a predicted peptidoglycan hydrolase with endopeptidase activity. RipA protein is secreted in culture filtrates by the TAT (twin-arginine translocation) system and has been shown to be one of the significant virulence factors in Mtb (Bhuwan et al., 2016). The signal peptide of RipA is predicted and cleavable signal sequence is confirmed experimentally (Malen et al., 2007). The MAP1203 gene was identified under the environmental conditions to be up-regulated more than 28-fold, and encodes for an important factor associated with the ability of MAP to interact with intestinal epithelial cells (Patel et al., 2006; Alonso-Hearn et al., 2010). Due to the presence of signal peptide of TAT secretion system in MAP1203 protein as well as significant homology (79%) to Mtb RipA secreted effector, it is highly likely that MAP1203 undergoes proteolytic cleavage and either it is translocated on the outer membrane or into the cytosol of the host cell; however, future investigation will be necessary to examine this possibility. Furthermore, MAP1203 contains the arginine-glycine-aspartic acid (RGD) peptide motif found in various studies to aid microorganisms to bind to and initiate the invasion process to host cells (Bomsel and Alfsen, 2003; Asokan et al., 2006; Zimmermann et al., 2010), and suggesting importance of this motif in the uptake of MAP by the intestinal mucosa. In this study, by overexpressing the wild-type MAP1203 gene in *M. smegmatis*, non-invasive mycobacteria, we demonstrate that MAP1203 enhances the pathogen's ability to bind and invade the MDBK epithelial cells. When the RGD domain was mutated, altering the MAP1203 binding site, bacteria did not become less invasive, ruling out that RGD region is involved in the binding or invasion. The fact is that enterocytes do not express fibronectin integrin molecules, the receptor for the RGD fibronectin-binding motif (Krammer et al., 2002), which supports an idea that binding to integrin molecules only happens on M cells but not on enterocytes. It is possible that MAP1203 protein also binds to M cells using the RGD motif rather than enterocytes. It is, however, challenging to test this hypothesis in the laboratory because of the difficulty to culture M cells. Our findings shows that MAP1203 binds to glyceraldehyde-3-phosphate dehydrogenase (GAPDH) on the surface of the bovine epithelial cells and the dihydropyrimidinase-related protein 2 also known as the collapsin response mediator protein 2 (CRMP-2). CRMP-2 is mainly expressed in epithelial cells and regulates the remodeling of the cytoskeleton by the microtubule assembly and trafficking (Yoneda et al., 2012). Phosphorylation of CRMP-2 as a downstream step of the RhoA and Rac1 GTPase activity has been linked with involvement and function in *Toxoplasma gondii* invasion (Na et al., 2013), suggesting that CRMP-2 may also play a role in MAP invasion. Several studies have demonstrated GAPDH localization on the cell surface, and it has been associated with the binding of several microorganisms by host cells initiating the signal transduction (Tristan et al., 2011; Oliveira et al., 2012).

MAP is efficient in binding and crossing the mucosal barrier in ruminants. Even more, it cross the mucosa without creating many symptoms in the infect animal, suggesting that the crossing of the mucosa is silent. In other to bypass common pathways of uptake and not trigger inflammatory response, the pathogen may use alternative route to infect the cells. The recognition of GAPDH by pathogens have been associate with quite uptake (Gozalbo et al., 1998), and supports the hypothesis that MAP needs to invade the mucosa of the intestine without facing phagocytic cells, until the infection become established.

One possible hypothesis why bacteria would exploit GAPDH is that by signaling through the docking of MAP1203 to GAPDH (probably using a different domain than RGD), MAP can regulate many different pathways associated with the translocation of the bacteria across the mucosal membrane. In fact, GAPDH activation is known to induce the phosphorylation of many proteins (Tisdale and Artalejo, 2007) and, thus, initiating signal transduction.

In this study, we show that the overexpression of MAP1203 in the natural host is associated with both attachment and invasion of epithelial cells. Although two complementary methods of the pathogen-host protein-protein binding demonstrated similar results, it is still possible that interaction between MAP1203 and host proteins are dependent on the experimental conditions. Future work and characterization of interaction of MAP1203 protein with the host cell receptor(s) will help us to understand the importance of MAP1203 expression in the milk environment, and will help to define the role in MAP virulence.

## Author contributions

JE performed experiments, wrote paper. LD performed experiments, wrote paper. LF performed experiments. LB designed studies, wrote paper, senior investigator.

### Conflict of interest statement

The authors declare that the research was conducted in the absence of any commercial or financial relationships that could be construed as a potential conflict of interest.
